# Tinea Capitis: Current Status

**DOI:** 10.1007/s11046-016-0058-8

**Published:** 2016-09-06

**Authors:** R. J. Hay

**Affiliations:** Dermatology Department, Kings College Hospital NHS Trust, London, SE9 3RS UK

**Keywords:** Tinea capitis, Dermatophytosis, Clinical features, Epidemiology, Treatment

## Abstract

Tinea capitis remains a common childhood infection in many parts of the world. Yet knowledge of the underlying pathogenetic mechanisms and the development of effective immunity have shown striking advances, and new methods of diagnosis ranging from dermoscopy to molecular laboratory tests have been developed even though they have not been assimilated into routine practice in many centres. Treatment is effective although it needs to be given for at least 1 month. What is missing, however, is a systematic approach to control through case ascertainment and therapy.

## Introduction

Tinea capitis is a common infection of the scalp hair caused by dermatophyte fungi and occurring predominantly in children [1]. Its clinical manifestations range from mild scaling with little hair loss to large inflammatory and pustular plaques with extensive alopecia. Although prevalent in many countries in the early twentieth century, it was brought under effective control in Europe and North America after the introduction of griseofulvin and concerted public health interventions, whereas it remained endemic in other regions. However, over the last 10–20 years, this situation has changed with the spread of organisms, in particular *Trichophyton tonsurans*, in the Americas, Europe and Africa.

### Pathological Change and Immunology

There is a spectrum of clinical reactions in tinea capitis that also reflects the pathological changes. In some patients, there is a pronounced inflammatory reaction, a feature often seen in zoophilic infections or those spread from animals to human; by contrast in others, particularly those with anthropophilic dermatophytosis spread from human to human, lesions are often non-inflammatory and persistent. It is still not clear whether this reflects the level of immunological responsiveness to these infections and the vast majority of those affected have no underlying predisposing illness. After transfer of fungal cells from one host to another, the first phase of epidermal or hair shaft invasion in tinea capitis consists of adhesion between fungal cells and keratinocytes. This has been shown to be a time-dependent process in which the invading arthrospore is attached to an underlying keratinocyte over a 2–3 h period before germination [2]. The process is accompanied by structural changes in the organism such as swelling of arthrospores and the expression of an extracellular fibrillar layer. After apposition to the hair shaft dermatophytes develop modified intercalary cells, known as penetrating organs, around which hair shaft invasion is centred.

Hair penetration by dermatophytes involves the production of proteases, some of which are inducible in the presence of amino acid residues as well as disruption of intercellular junctions due to hyphal turgor pressure. Dermatophytes produce a variety of proteolytic enzymes, which work in acid, alkali or neutral environments [3], and these protease genes are variably expressed with distinct enzyme patterns being found in infections versus cultured conditions [4]. In *Microsporum canis*, at least three genes, *SUB1*, *SUB2* and *SUB3*, have been identified encoding serine proteases associated with infection [5]. Proteases with keratinase activity from certain dermatophytes are inducible by low-molecular-weight peptides released from the epidermal cells by the action of other fungal proteinases [4]. Other enzymes such as sulphur transporters are also involved.

Generally, the fungi are prevented from invading human skin in a number of different ways. The expression of naturally produced antimicrobial peptides, including human β-defensins (hBDs), cathelicidin LL-37 and dermicidin [6], provides a major source of innate defence. These peptides are known to have activity against bacteria, viruses and fungi and play a key role in protection against skin infections including dermatophytes as well as *Candida albicans*. *Microsporum canis* infection has also been shown to trigger rapid secretion of IL-1β both in vitro and in vivo, and this is dependent on the activation of inflammasomes, intracellular multiprotein complexes that control the release of Il-1β [7]. Other innate inhibitory mechanisms, important in the scalp, include medium chain length fatty acids in sebum which are inhibitory to dermatophyte growth [8]. This activity appears to reside in saturated fatty acids with chain lengths of 7, 9, 11 and 13 carbon residues. The mechanism is thought to underlie the observation that tinea capitis is largely seen in children, as at puberty, the relative composition of different unsaturated fatty acids in sebum is altered. Killing of dermatophytes by both murine and human neutrophils and macrophages can also be demonstrated [9]. Human neutrophils have been shown to destroy up to 60 % of dermatophyte germlings within 2 h; macrophages kill up to 20 % in a similar time.

In a mouse model of dermatophytosis using the natural murine pathogen infecting hair, *Trichophyton mentagrophytes* (the original isolate was the *quinckeanum* variant), transfer of T-lymphocytes bearing the Thy-1 helper phenotype from immune animals to naive recipients is the key event in determining immunity. During primary infection in mice, there is evidence of polyclonal suppression of mitogen-induced lymphocyte activation during the phase of activation of lymphocytes reactive with dermatophyte antigens [10]. Immunity to infection can be transferred to irradiate naive animals with lymphocytes bearing the Thy-1 phenotype, but not Ly-2.2 [11]. Passive transfer of antibody will also not convey resistance on recipients. Using a new model of dermatophyte infection in mice, it has been shown that there was a specific cytokine profile characterized by the overexpression of transforming growth factor-ß, interleukin (IL)-1ß and IL-6 mRNA during infection, suggesting a role of the T-helper 17 pathway [12]. Hence, T cell activation is a key event in limiting the infection and this provides support for the possibility of immunization.

Studies of human populations using molecular techniques have identified a number of genes which appear to be associated with susceptibility to tinea capitis. These include macrophage regulators as well as some concerned with keratin expression, leucocyte activation and migration, extracellular matrix integrity and remodelling, epidermal maintenance and wound repair, as well as cutaneous permeability [13].

In man, there is a correlation between inflammatory responses, T-lymphocyte activation, and recovery. In tinea capitis, the development of delayed-type hypersensitivity and presumed T cell mediated immunity to dermatophyte antigen correlates with recovery from the infection [14]. The possibility that dermatophytes interfere with the process of immunological activation in the skin is supported by immunohistochemical studies of biopsies from chronically infected skin. In acute dermatophyte infections, immunophenotypic techniques demonstrate the presence of effector lymphocytes in the vicinity of the infection [15]. Work has now shown that the dermal infiltrate mainly contains cells which are Leu2a positive (viz. T-helper cells) [15]. There is also evidence of down-regulation of other skin markers including adhesion factors such as ICAM-1 [16].

### Epidemiology

Those species of dermatophyte fungi most likely to cause tinea capitis vary from geographic region to region. In turn, this pattern may change with time, particularly as new organisms are introduced by migration or immigration [1, 17, 18]. In tinea capitis, anthropophilic species predominate in many countries as the main causes of infection. The principal changes in the epidemiology of tinea capitis in recent years has been the rise of *M. canis* as the dominant organism in infections in some parts of Europe [18] and the spread of *T. tonsurans* in urban communities in the USA [1] and Western Europe, e.g. the UK [18] and France [19]. In addition, *T. tonsurans* has spread to both South America [20] and West Africa [21]. There is also spread of *T. tonsurans* in Far Eastern countries such as Japan although this has mainly been associated with infection in older children and adults and associated with wrestling [22]. Because of the association between trends in tinea capitis and movement of populations, there have also been increases in infections due to *Trichophyton violaceum* and *Microsporum audouinii* in countries that were previously not endemic for these organisms [23, 24]. However, the rise in tinea capitis due to *T. tonsurans* has presented a challenge to both treatment and community infection control and in most areas. There is little evidence that the rise in the prevalence of this infection has been halted, although there is some data to show that this epidemic may be declining in the Western USA [25].

### Clinical Varieties of Infection

Many species of dermatophyte are capable of invading hair shafts, but some (e.g. *T. tonsurans, Trichophyton schoenleinii* and *T. violaceum*) have a predilection for this pattern of infection, whereas *Epidermophyton floccosum* and *Trichophyton concentricum* do not cause tinea capitis. All dermatophytes causing scalp ringworm can invade smooth or glabrous skin, and some can penetrate nails as well, e.g. *T. soudanese*. There are three main types of hair shaft invasion that, in part, determine clinical presentation [1, 26].

- *The ectothrix form* In this type of infection, the hair shaft is invaded at the level of mid-follicle. The intrapilary hyphae grow down towards the bulb of the hair. The common causes are *Microsporum* species, but *Trichophyton verrucosum* can cause a form of ectothrix infection—the arthroconidia are larger. Fluorescence under filtered ultraviolet or Wood’s light is characteristically present in most ectothrix infections caused by *Microsporum* species. In terms of clinical appearance, ectothrix infections are usually scaly and often inflamed. There is hair loss with hair shafts breaking 2–3 mm or more above the scalp level.
- *The endothrix form* The endothrix type of infection may be caused by *T. tonsurans, Trichophyton soudanense* and members of the *Trichophyton rubrum* of African origin group, *T. violaceum,* or *T. rubrum* (rare). This type of infection is non-fluorescent under Wood’s light. Hairs often break at scalp level leaving swollen hair stubs within the follicles (black dot ringworm).
- *Favus* The favic type of infection is caused by the anthropophilic dermatophyte *T. schoenleinii.* The affected hairs are less damaged than in the other types, and may continue to grow to considerable lengths. Air spaces in the hair shafts are characteristic and fungal hyphae form large clusters at the base of hairs where they enter the follicle at the level of the epidermis.

### Clinical Features

The clinical appearance of ringworm of the scalp is variable, depending on the type of hair invasion, the level of host resistance and the degree of inflammatory host response. Most affected patients are children for 6 months to 10–12 years of age. Tinea capitis can sometimes occur in adults and in this case is usually caused by anthropophilic fungi. The pattern varies from a few, broken-off hairs with little scaling, detectable only on careful inspection, to a severe, painful, inflammatory mass or kerion covering most of the scalp. Itching is variable. In all types, the characteristic features are partial hair loss with some degree of inflammation.

In *M. audouinii* infections, the basic lesions are patches of alopecia, often circular in shape, but showing numerous broken-off hairs. Inflammation is minimal, but fine scaling is characteristic. There may be several or many such patches arranged more or less randomly. In *M. canis* infections, the most common *Microsporum* species causing tinea capitis, the pattern is similar but lesions are generally more inflamed and itchy. In *T. tonsurans* and *T. violaceum* infections, a minimally inflammatory type of patchy hair loss occurs. Formation of black dots (swollen hair shafts) as the affected hair breaks at the surface of the scalp is classical in this condition, but may be hard to find as they can be sparsely distributed. The patches, which are usually multiple, may show minimal scaling, sometimes mimicking discoid lupus erythematosus, sometimes seborrhoeic dermatitis. Other forms include diffuse and patchy alopecia, even with involvement of single isolated hair shafts and without scales. The common clinical types are known as: grey patch (scaling with patchy hair loss), black dot and diffuse alopecia. But on occasions, severely inflammatory and raised lesions can occur.

The most severe pattern of reaction is known as a kerion. It is a painful, inflammatory mass in which those hairs that remain are loose. Follicles discharge pus. The area affected is usually limited, but occasionally, a large confluent may involve much of the scalp. Lymphadenopathy is frequent. This reaction is usually caused by one of the zoophilic species, typically *T. verrucosum* or *T. mentagrophytes*, but occasionally, anthropophilic infections may suddenly become inflammatory and develop into kerions. Generally, however, pustule formation represents an inflammatory response to the fungus itself rather than a secondary bacterial infection. If secondary bacterial infection occurs, it is usually present under crusts covering the inflammatory mass and removal of these is an important part of management.

The attack rate for epidemic infections caused by anthropophilic species may be as high as 40 %, within school classes in some areas of sub-Saharan Africa for example, but it is usually lower than this.

Infection with *T. schoenleinii* (favus) is now seen rarely and sporadically in a variety of countries, such as Ethiopia, where it is still endemic, as well as rare cases elsewhere. The classical picture of favus is characterized by the presence of yellowish cup-shaped crusts containing hyphae and known as scutula which form at the base of hair shafts where they emerge from the skin. Adjacent scutula enlarge to become confluent and form a mass of pale powdery crusts. Many patients may show less distinctive changes, in early cases, usually no more than perifollicular redness and some matting of the hair. Extensive, patchy hair loss with scarring alopecia and atrophy scattered between patches of normal hair growth may be found in long-standing cases; here, much of the hair loss is irreversible. Although the initial infection occurs in childhood, it often fails to clear spontaneously at puberty, particularly in women.

### Differential Diagnosis

The differential diagnosis of tinea capitis includes all conditions capable of causing patchy baldness with inflammatory changes of the scalp. Alopecia areata may show erythema, and although of itself, it is not a scaly condition. Seborrhoeic dermatitis is usually more diffuse than tinea capitis. Discoid lupus erythematosus, lichen planus and other causes of scarring alopecia may sometimes have to be considered.

### Complications

Secondary bacterial infection is not common even in kerion, where this may occur under large superficial crusts (see above) rather than in the form of folliculitis.

A phenomenon sometimes seen with inflammatory tinea capitis such as kerion is the emergence of a secondary rash, usually small follicular papules in other areas of the body such as the trunk or limbs. Rarely, erythema nodosum has been described in association with tinea capitis. These reactions are known as id reactions [27], and they are thought to represent an immune complex deposition reaction.

### Laboratory Diagnosis

Until recently, the means of laboratory diagnosis in tinea capitis has been the combined use of direct microscopy as well as culture, the final report being received up to 2 weeks after obtaining a sample. Samples are taken by scraping or by using a scalp brush such as a disposable toothbrush or swab. This is still the practice in most laboratories [1]. However, the introduction of molecular techniques using different forms of polymerase chain reaction (PCR) screening [28] has begun to result in the development of faster and accurate ways of identifying dermatophytes. A recent study, for instance, has compared two different molecular techniques—multiplex ligation-dependent probe amplification (MLPA) and rolling circle amplification (RCA)—for rapid detection of infection of the scalp with great accuracy as well as speed [29].

Another adaptation of a diagnostic technique widely used in dermatology has been to employ the dermatoscope for close inspection of the scalp [30]. Although, not as yet subject to comparative study, there are visual features of the scalp in infected areas that may be distinctive for different organisms. For instance, in infections caused by *T. tonsurans,* the infected areas showed multiple comma-shaped hairs, whereas with *M. canis,* the scalp with dermoscopy shows dystrophic and elbow-shaped hairs, and in addition, there are different height levels of broken hair [31]. These studies need wider validation but may provide a rapid provisional diagnosis, sufficient to initiate treatment.

### Management

Topical antifungal therapy has little place in the management of tinea capitis except as an adjunct to oral therapy [1, 32]. There is evidence that final relapse rates of infection following topical therapy are high, although the clinical appearances and itching may initially improve.

Treatment for tinea capitis relies on the use of terbinafine, itraconazole, griseofulvin and fluconazole [1, 32]. There is no clinical evidence to support the use of other oral antifungals, including the newer azoles such as voriconazole or posaconazole. Griseofulvin was the first effective drug used of the treatment of tinea capitis and is still widely used in resource-poor settings as it remains effective. It is useful particularly for *Microsporum* infections, but it is not available in paediatric form (liquid or small tablet sizes) in many countries. Although massive single-dose therapy with griseofulvin and intermittent dose regimens (25 mg/kg twice a week) have had some success, in general, conventional daily therapy is advisable (10–15 mg/kg). In small-spored ectothrix infections, griseofulvin for at least 6 weeks is usually adequate. In some infections such as those caused by *T. tonsurans* and *T. schoenleinii* infections, much longer courses and sometimes higher dosage (20 mg/kg/day) of griseofulvin therapy may be needed.

In addition, both itraconazole and terbinafine are now licensed in some countries for use in children, and are alternatives for certain infections such as those caused by *Trichophyton* species, although there are fewer data and, in the case of terbinafine, the drug appears to be less effective in disease caused by *Microsporum* species. The best length of treatment for *T. tonsurans* and *T. violaceum* infections with terbinafine appears to be 1 month. There is some evidence that higher doses (double the standard dose) of terbinafine may be more effective for *Microsporum*. The appropriate length of treatment with either itraconazole or fluconazole is not established, although both appear to be effective against *T. tonsurans*. Usually, one month of treatment is given. Dosing regimens for tinea capitis are highlighted in the below table.

**Table:** Dosing regimens for tinea capitis

Terbinafine	<10 kg 62.5 mg, 10–20 kg 125 mg, >20 kg 250 mg—all daily 4 weeks
Itraconazole	2–4 mg/kg/day for 4–6 weeks
Griseofulvin	10 mg/kg 6–8 weeks (20 mg/kg considered in some *T. tonsurans* and *T. schoenleinii* infections)

The most recent Cochrane review of the oral treatment of tinea capitis [33] concluded as follows: “The best evidence suggests that newer treatments including terbinafine, itraconazole and fluconazole may be similar to griseofulvin in children with tinea capitis caused by *Trichophyton* species. Newer treatments may be preferred because shorter treatment durations may improve treatment adherence, although they may be more expensive”. A more recent systematic review using additional published data suggested that for *Trichophyton* infections of the scalp, terbinafine was preferable, whereas griseofulvin was favoured in *Microsporum* infection [34]. The policy in our department is to follow this approach.

For the treatment of kerions of the scalp, careful removal of crusts (stratum corneum and dried serous exudates) using wet compresses is useful as it allows healing, and the possibility of coexisting bacterial infection under the crusts should be considered. If confirmed by culture, antibiotics should be given. In general, kerions are often less painful than their inflammatory appearance suggests. Occasionally, in children with extensive kerions, it may be easier to remove the crusts initially in a hospital outpatient setting. Permanent hair loss from scarring is usually less than would be expected as hairs frequently grow back even in an extensively infected area.

In severely inflammatory forms, there have been some arguments in favour of using oral steroids to inhibit the inflammatory response. But generally it is best review all cases early after starting antifungal therapy, and only use oral steroids in severe cases with widespread id reactions.

Ketoconazole shampoo or selenium sulphide is used 2–3 times weekly to prevent spread in the early phases of therapy, in combination with an oral treatment. A similar approach is used for siblings of patients with anthropophilic tinea capitis after careful inspection including taking scalp cultures to ensure that they are not infected. If they are positive on culture but the scalp is clinically normal, they are described as carriers [35] and most advise treating them with antifungal shampoos as described above. Opinion remains divided over the correct approach to siblings of children known to be infected with *T. tonsurans* and who have positive cultures on scalp brushing. It is the policy in our department to treat them as infected using oral therapy because many show subclinical or limited hair shaft infections.

If the patient has a zoophilic infection, it is important to identify and treat the animal source. The culture or molecular identification of the pathogen should provide a clue to the most likely culprit.

### Community Control of Tinea Capitis

Tinea capitis was once regarded in Europe and the USA as a mark of poverty and destitution and therefore an illness to be combated. Until the discovery of griseofulvin, a series of public health measures such as exclusion from school, mass treatment with topical and radiation therapy were promoted. However, with the appearance of griseofulvin, a concerted treatment campaign for affected children and identification of cases was instituted throughout Europe and the USA and the infection was brought under control as an endemic problem. The endemic infections were never addressed comprehensively as a public health issue in resource-poor settings in the tropics.

The situation has changed with the spread of *T. tonsurans*. Because of its apparent propensity to spread in children with black hair type, the infection has been found widely in the USA and Canada before becoming established in the Caribbean, some European communities (e.g. the UK and France), South America and now West and East Africa. This outbreak has not been matched with the public reaction or proactive public health measures, and as a result, the infection has continued to spread.

## Conclusion

Tinea capitis is still a common infection with prevalence rates exceeding 40 % in some communities [36, 37], whereas even 50 years ago, there was a public and governmental will to find adequate control measures; this infection is no longer regarded as a health priority. Control of tinea capitis is not impossible, and with current understanding of the immunology and host susceptibility, including the latest findings of specific CARD 9 gene mutations associated with widespread deep dermatophytosis [38], it is theoretically possible to develop an immunizing antigen. However, this approach remains a long way off, and at present, increased surveillance in schools coupled with prompt treatment of cases with antifungals is the best approach to limit continued spread of infection.
